# Maternal supplementation of seaweed-derived polysaccharides improves intestinal health and immune status of suckling piglets

**DOI:** 10.1017/jns.2015.16

**Published:** 2015-08-24

**Authors:** G. Heim, J. V. O'Doherty, C. J. O'Shea, D. N. Doyle, A. M. Egan, K. Thornton, T. Sweeney

**Affiliations:** 1 School of Agriculture and Food Science, University College Dublin, Belfield, Dublin 4, Republic of Ireland; 2 School of Veterinary Medicine, University College Dublin, Belfield, Dublin 4, Republic of Ireland

**Keywords:** Cytokines, Intestinal morphology, Microbiota, Piglets, Seaweed-derived polysaccharides, BW, body weight, CD, crypt depth, cDNA, complementary DNA, *C*_*t*_, cycle threshold, *FABP2*, fatty acid binding protein 2, FOXP3, forkhead box P3, GCN, gene copy number, GIT, gastrointestinal tract, *GLUT1*, glucose transporter 1, HMBS, hydroxymethyl-bilane synthase, *IFN-γ*, interferon *γ*, LPS, lipopolysaccharide, *PEPT1*, peptide transporter 1, PPIA, peptidylprolylisomerase A, RT-qPCR, real-time PCR, SDP, seaweed-derived polysaccharide, *SGLT1*, sodium–glucose-linked transporter 1, TGF-*β1*, transforming growth factor *β*1, VH, villus height

## Abstract

The experiment investigated the effect of maternal dietary supplementation of seaweed-derived polysaccharides (SDP) (–SDP *v.* +SDP, *n*   20) from day 83 of gestation until weaning (day 28) on selected sow faeces and piglet digesta microbiota populations, piglet small-intestinal morphology, and intestinal nutrient transporter and inflammatory cytokine gene expression at birth, 48 h after birth and weaning. The effect of maternal dietary treatment on the piglet gene expression profile of inflammatory cytokines in the colon following a lipopolysaccharide (LPS) challenge was also investigated. Dietary SDP reduced sow faecal Enterobacteriaceae gene numbers at parturition. Small-intestinal morphology, nutrient transporter and cytokine gene expression in newborn piglets did not differ between maternal dietary treatments (*P* > 0·10). At 48 h after birth, sodium–glucose-linked transporter 1 gene expression was down-regulated in the ileum of piglets suckling the SDP-supplemented sows compared with those suckling the basal sows (*P* = 0·050). There was a SDP × LPS challenge interaction on *IL-1* and *IL-6* gene expression in the colon of piglets (*P* < 0·05). The gene expression of *IL-1* and *IL-6* was down-regulated in the LPS-challenged colon of piglets suckling the SDP sows compared with those suckling the basal sows (*P* < 0·05). However, there was no difference in *IL-1* and *IL-6* gene expression in the unchallenged colon between treatment groups. At weaning, piglets suckling the SDP-supplemented sows had increased villus height in the jejunum and ileum compared with those suckling the basal-fed sows (*P* < 0·05). In conclusion, maternal dietary SDP supplementation enhanced the immune response of suckling piglets and improved gut morphology, making them more immune competent to deal with post-weaning adversities.

Piglets are born in an agammaglobulinaemic state^(^^1^^)^, due to the six-layered placenta of the dam^(^^2^^)^. The gastrointestinal tract (GIT) of the piglet encounters numerous challenges at birth and weaning^(^^3^^)^. Immediately after birth, the neonatal GIT is rapidly colonised by bacteria originating from the mother or the environment^(^^4^^)^. It has become clear that this primary colonisation is important for the right development and programming of the animal's local and systemic immune system^(^^5^^)^. Furthermore, the GIT takes complete responsibility for the provision of nutrients, and shifts from processing amniotic fluid swallowed during and after birth to a nutrient-enriched colostrum ingested after birth^(^^3^^)^. At weaning, pigs are exposed to a large number of stressors, such as separation from the dam, transition from milk to a diet based on plant polysaccharides^(^^6^^)^, and withdrawal of maternal IgA from milk that acts locally in the intestine of suckling piglets^(^^7^^)^. These factors combined can disturb the intestinal immune system and microbiota equilibrium^(^^8^^)^, which contribute toward a decrease in daily gain immediately post-weaning. In order to adapt to the changes encountered at birth and weaning, the GIT undergoes accelerated tissue growth (birth) and functional maturation (birth and weaning)^(^^9^^)^, characterised by increases in intestinal weight and length, villus height (VH) and crypt depth (CD), cell migration rate, RNA and DNA contents, and the adaptation of intestinal enzymic activity^(^^10^^)^. The intestinal epithelium is not only a physical barrier and a nutritional site; it also acts as a crucial regulator of intestinal immune homeostasis^(^^11^^)^. In response to bacteria colonisation at birth and weaning, the enterocytes can act as a component of the immune system by secreting cytokines^(^^12^^,^^13^^)^.

Recent research has indicated that supplementing sows with seaweed-derived polysaccharides (SDP), containing laminarin and fucoidan, bioactives containing antimicrobial, prebiotic and immunomodulatory properties, from day 107 of gestation influenced piglet immune status at weaning, and had beneficial effects on weaning-associated intestinal dysfunction and growth depression immediately after weaning^(^^14^^–^^18^^)^. However there is no information about the effect of maternal dietary treatment of SDP on intestinal dysfunction immediately after birth and after ingestion of colostrum. Furthermore, Leonard *et al*.^(^^17^^)^ found no effect of maternal SDP supplementation on piglet body weight (BW) at birth. The starting date of the supplementation (day 107 of gestation)^(^^17^^)^ may have been too late to promote an effect on piglet birth weight. Studies have been shown that the nutrient needs for fetal growth increase from day 69 of gestation^(^^19^^)^, and the transfer of nutrients to the fetus increases between days 90 and 100 of gestation^(^^20^^)^.

Thus, the objective of the present study was to investigate the effect of maternal dietary supplementation of SDP from day 83 of gestation until weaning (day 28) on piglet BW at birth, and small-intestinal morphology, nutrient transporter and inflammatory cytokine gene expression, and colonic microbiota population of piglets at birth (0 h), 48 h after birth and weaning. The effect of maternal dietary treatment on the piglets’ gene expression profile of inflammatory cytokines in the colon following a lipopolysaccharide (LPS) challenge was also investigated. The hypothesis of this study is that maternal SDP supplementation from day 83 of gestation would improve piglet BW at birth and modulate selected intestinal microbial populations, the inflammatory response and aspects of intestinal health of piglets at birth and during the suckling period, making them more immune competent to deal with post-weaning adversities.

## Materials and methods

All procedures described in the present experiment were conducted under experimental license from the Irish Department of Health in accordance with the Cruelty to Animals Act 1876 and the European Communities (Amendments of the Cruelty to Animals Act, 1876) Regulations.

### Experimental design and dam diets – gestation and lactation period

A total of twenty crossbred pregnant gilts (Large White × Landrace genetic lines; Hermitage) were randomly assigned to one of the two dietary treatments (ten gilts/treatment): (T1) basal gestation/lactation diet (control) and (T2) basal gestation/lactation diet supplemented with 10·0 g SDP/d from day 83 of gestation until weaning (day 28). The quantity of SDP (Bioatlantis Ltd) used was based on previous work by Leonard *et al*.^(^^17^^)^. The SDP supplement (10·0 g/d) contained laminarin (1·0 g), fucoidan (0·8 g) and ash (8·2 g), and was extracted from a *Laminaria* spp. according to the procedure described by Lynch *et al*.^(^^21^^)^. The gestation diet contained 140 g/kg of crude protein (CP), 13·5 MJ/kg of digestible energy (DE) and 4·4 g/kg of standardised ileal digestible (SID) lysine. The lactation diet contained 190 g/kg of CP, 14·5 MJ/kg of DE and 8·5 g/kg of SID lysine. All amino acid requirements were met relative to lysine^(^^22^^)^. The ingredient composition and chemical analysis of the diets are given in Table 1.

**Table 1.** Ingredients and chemical composition of the experimental diets (g/kg, unless otherwise indicated)

	Gestation diet*	Lactation diet*
Composition		
Wheat	303·8	352·5
Barley	300·0	300·0
Soyabean meal	67·0	182·0
Dried maize distillers grains	60·0	100·0
Soya hulls	70·0	
Beet pulp	100·0	
Soya oil	70·0	30·0
Vitamins and minerals†	3·0	2·5
Salt	3·0	5·0
Dicalcium phosphate	11·2	12·0
Limestone	12·0	12·0
Lysine HCl		2·0
dl-Methionine		1·0
l-Threonine		1·0
Analysis of chemical composition		
DM	870·9	873·2
Crude protein (N × 6·25)	140·0	190·0
Gross energy (MJ/kg)	16·9	17·1
Ash	55·2	57·1
Digestible energy (MJ/kg)‡	13·5	14·5
Lysine‡	5·5	10·0
Methionine and cysteine‡	3·3	6·0
Threonine‡	3·85	7·0
Tryptophan‡	1·0	1·8
Ca‡	8·7	9·3
P‡	5·0	5·2

* T1 = basal diet; T2 = basal diet supplemented with 10·0 g seaweed-derived polysaccharides containing laminarin and fucoidan/d.

† Gilt/sow diet provided (per kg diet): 250 mg choline chloride; 140 mg Fe; 120 mg Zn as ZnO; 67 mg α-tocopherol; 47 mg Mn as MnO; 25 mg Cu as CuSO_4_; 12 mg nicotinic, acid; 10 mg pantothenic, acid; 4 mg phytylmenaquinone; 2 mg riboflavin; 2 mg thiamin; 1·8 mg retinol; 0·6 mg iodine as calcium iodate on a calcium sulphate/calcium carbonate carrier; 0·3 mg Se as sodium selenite; 0·025 mg cholecalciferol; 0·015 mg pyridoxine; 0·01 mg cyanocobalamin.

‡ Calculated from amino acid and digestible energy values of ingredients^(^^67^^)^.

Prior to day 83 of gestation, the gilts were housed in groups of ten. From day 83 until day 106 of gestation, they were housed individually in crates (2·0 × 0·6 m) in the gestation house. In the farrowing house, the gilts were housed individually in farrowing pens (2·2 × 2·4 m). The gestation and farrowing houses were maintained at 20°C throughout the experiment.

The experimental supplement (SDP) was top-dressed on the gestation diet and added to the trough prior to feeding the lactation diet each morning (09·00 hours) to ensure consumption. The dams received specific amounts of feed in the following quantities: 2·5 kg/d of gestation diet from day 83 until day 106 of gestation. They were fed 2·0 kg/d of lactation diet from day 107 of gestation until the day of farrowing and then the feed supply was increased by 1·0 kg/d until day 3 post-farrowing and by 0·5 kg/d until day 6 post-farrowing. Afterwards, the sows were allowed semi-*ad libitum* consumption of the diet, which was adjusted for each sow depending on daily intake. The sows were fed in three equal meals provided at 09·00, 13·00 and 17·00 hours. They had *ad libitum* access to drinking water throughout the experimental period.

On the expected farrowing date, fresh sow faecal samples (approximately 10 (sd 1·0) g) were collected from the ground into sterile containers (Sarstedt) and stored at −20°C for quantification (microbial genomic DNA analysis) of *Lactobacillus* spp. and Enterobacteriaceae.

### Management of piglets and collection of colostrum samples

All farrowings were supervised. At parturition, piglets (Meat line boars × Large White × Landrace genetic lines gilts) were individually weighed and ear-tagged. Between 6 and 12 h after the birth of the last piglet, litter size was adjusted by cross-fostering piglets within sow dietary treatments to ensure that sows nursed a similar number of piglets (*n* 12 piglets/sow), and this was maintained throughout the suckling period. Cross-fostering performed on average at 20 h after birth has no adverse effects on growth performance^(^^23^^)^ and IgG serum concentration^(^^2^^)^ of both adopted and biological piglets. The individual piglet BW was recorded at birth and weaning and the average daily gain calculated from these data. The piglets received an intramuscular injection of Fe-dextran (Ferdex 100; Medion Farma Jaya) on day 7 after birth. No creep feed was offered to the piglets throughout the lactation period, and piglets did not have access to the sows’ feed.

During parturition, approximately 30 ml of colostrum sample was collected from each sow after hand-milking the first pair of mammary glands. To facilitate colostrum sampling, piglets were removed from the sow, and milk ejection was induced after administration of 1 ml of oxytocin (Pitocina, Watson Laboratories Inc.) into the marginal ear vein of the sow. The colostrum samples were immediately frozen at −20°C before analysis for IgG concentration.

A diarrhoea score was recorded daily on litter basis from birth to weaning using a scale from 0 to 3: (0) no diarrhoea; (1) slight; (2) middle; (3) acute^(^^24^^)^. Diarrhoea score was performed by one trained person with no prior knowledge of the dietary treatment of the sows.

A total of sixteen piglets (*n* 8 piglets/treatment group, one piglet/sow) were selected at birth, with an average BW of 1·3 (sd 0·3) kg. The selection was based on the parturition of the third piglet. One trained operator was positioned at the back of the sow from the initiation of parturition, in order to make sure that the third piglet would not touch anything other than the sow vaginal tract and the gloved hand of the operator.

A total of sixteen piglets were selected at 48 h after birth (*n* 8 piglets/treatment group, one piglet/sow), with an average BW of 1·8 (sd 0·1) kg.

A total of sixteen piglets were selected at weaning (*n* 8 piglets/treatment group, one piglet/sow), with an average BW of 7·4 (sd 0·9) kg. The selection of piglets at 48 h after birth and weaning was based on the litter average BW.

The piglets were humanely killed on each day (immediately after birth (0 h), at 48 h after birth and at weaning) by lethal injection with pentobarbital sodium (Euthatal Solution, 200 mg/ml; Merial Animal Health Ltd) at a rate of 0·71 ml/kg BW to the cranial vena cava, and the entire intestinal tract was immediately removed.

### Chemical analysis

The feed samples were milled through a hammer mill provided with a 1-mm screen (Christy and Norris hammer mill; Christy Turner Ltd). The gestation and lactation feed samples were analysed for N, DM, ash and gross energy as described by Heim *et al*.^(^^25^^)^. The feed and dried faeces samples were milled through a hammer mill provided with a 1-mm screen (Christy and Norris). The DM of dried faeces and feed was determined after drying overnight at 103°C. Ash was determined after ignition of a known weight of concentrates or faeces in a muffle furnace (Nabertherm) at 500°C. The N content of both feed and faeces was determined using the LECO FP 528 instrument (Leco Instruments, U.K Ltd). Neutral-detergent fibre was determined using a Fibertec extraction unit (Tecator). The gross energy of the feed and faeces was determined using a Parr 1201 oxygen bomb calorimeter (Parr). The total laminarin content of the SDP supplement was determined using a Megazyme kit (Megazyme International Ireland Limited). Fucoidan levels were determined using the method of Usov *et al.*^(^^26^^)^.

### Collection of piglet tissue and digesta samples at birth (0 h), 48 h after birth and weaning

Immediately after slaughter, the entire intestinal tract was removed by blunt dissection and sections of the duodenum (10 cm from the stomach), jejunum (60 cm from stomach) and ileum (15 cm from caecum) were excised and fixed in 10 % phosphate-buffered formalin for VH and CD measurements. Ileum and colon tissues (second loop of the proximal colon) were excised, emptied by dissecting them along the mesentery and rinsed using sterile PBS (Oxoid). Two tissue sections of 1 cm^2^, which had been stripped of the overlying smooth muscle, were cut from each tissue. Then, one section from each tissue was placed in 1 ml of Dulbecco's modified Eagle's medium (Gibco, Life Technologies) in the presence or absence of bacterial LPS (source: *Escherichia coli* strain B4; Sigma Aldrich Ireland Limited) at a concentration of 10 mg/ml. Both LPS-challenged and unchallenged tissues were incubated at 37°C for 120 min before being removed, blotted dry and weighed. Approximately 1–2 g of the ileum and colon tissues were cut into small pieces and stored in 15 ml RNAlater^®^ solution (Applied Biosystems) overnight at 4°C. RNAlater^®^ was then removed before storing the samples at −80°C.

Colon digesta samples (approximately 2 g) were collected into sterile containers (Sarstedt) and stored at −20°C for quantification (microbial genomic DNA analysis) of *Lactobacillus* spp. and Enterobacteriaceae.

### Colostrum IgG quantification

An assay for colostrum concentration of IgG was performed using a specific pig-ELISA IgG quantification kit (Bethyl Laboratories Inc.). For colostrum sample preparation, 10 ml of colostrum were centrifuged (15 min at 2500 ***g***; Rotanta 460 R; Hettich Lab Technology) and 10 µl of the defatted fraction containing the IgG were added into 990 µl of 1× Dilution Buffer B to give a 1:100 dilution. The serial dilutions were repeated until the dilution recommended for colostrum: 1:1 000 000. The IgG was quantified according to the manufacturer's instructions.

### Microbiology – DNA extraction and quantitative real-time PCR

Microbial genomic DNA was extracted from sow faeces and piglet colon digesta samples using a QIAamp DNA stool kit (Qiagen) in accordance with the manufacturer's instructions. Quantity and quality of DNA were assessed using a NanoDrop Spectrophotometer (ND1000, Thermo Scientific). Standard curves were prepared as described by O'Shea *et al.*^(^^27^^)^. Briefly, genomic DNA from all samples was pooled and amplified through routine quantitative real-time PCR (RT-qPCR) using *Lactobacillus* spp. and Enterobacteriaceae primers. The primer sequences were as follows: *Lactobacillus* spp. – forward 5′–AGCAGTAGGGAATCTTCCA–3′ and reverse 5′–CACCGCTACACATGGAG–3′, 58°C; 341 bp and Enterobacteriaceae – forward 5′–CATTGACGTTACCCGCAGAAGAAGC–3′ and reverse 5′–CTCTACGAGACTCAAGCTTGC–3′, 58°C; 190 bp^(^^28^^)^. All primers were designed using Primer Express^™^ Software (Applied Biosystems) and synthesised by MWG Biotech. Serial dilutions of these amplicons served to generate standard curves using RT-qPCR (ABI 7500 Real-Time PCR System; Applied Biosystems Ltd) permitting estimations of absolute quantification based on gene copy number (GCN)^(^^29^^)^. The RT-qPCR were performed in a final reaction volume of 20 µl containing 2 µl template DNA, 1 µl of forward (100 pm) and 1 µl of reverse primers (100 pm), 10 µl SYBR Green PCR Master Mix (Applied Biosystems) and 6 µl nuclease-free water. The thermal cycling conditions involved an initial denaturation step at 95°C for 10 min followed by forty cycles of 95°C for 15 s and 65°C for 1 min. Dissociation analyses of the RT-qPCR product were performed to confirm the specificity of the resulting RT-qPCR products. All samples were prepared in duplicate. The mean cycle threshold (*C*_*t*_) values of duplicates of each sample were used for calculations.

### Small-intestinal morphology

The preserved segments (duodenum, jejunum and ileum) were prepared using standard paraffin-embedding techniques. The samples were sectioned at 5 µm thickness and stained with haemotoxylin and eosin^(^^30^^)^. Measurements of fifteen well-orientated and intact villi and crypts were taken for each segment. The VH and the CD were measured as described by Heim *et al*.^(^^25^^)^. The results are expressed as mean VH or CD in μm. The VH:CD ratio was calculated.

### Nutrient transporter and inflammatory cytokine gene expression – RNA extraction, complementary DNA synthesis and quantitative real-time PCR

RNA was extracted from approximately 50 mg of ileum and colon tissue samples using the GenElute Mammalian Total RNA Miniprep Kit (Sigma Aldrich Corporation) according to the manufacturer's instructions. The total RNA was quantified using 1·5 µl of total RNA on a NanoDrop Spectrophotometer (ND1000; Thermo Scientific) and samples with a 260:280 ratio ≥2·0 were considered suitable for complementary DNA (cDNA) synthesis. Total RNA integrity (i.e. quality and quantity) was assessed by analysing 1 µl of total RNA using the Agilent 2100 Bioanalyser version A.02.12 (Agilent Technologies, Inc.) using RNA Nano LabChips^®^ (Caliper Technologies Corporation). The cDNA synthesis was performed using 1 µg of total RNA and oligo(dT)_20_ primers in a final reaction volume of 20 µl using a Superscript^™^ III First-Strand synthesis kit (Invitrogen, Life Technologies) following the manufacturer's instructions. The final reaction volume of 20 µl was then adjusted to 250 µl using nuclease-free water. All primers for the selected nutrient transporter (peptide transporter 1 (*PEPT1/SLC15A1*), sodium–glucose-linked transporter 1 (*SGLT1/SLC5A1*), glucose transporter 1 (*GLUT1/SLC2A1*), *GLUT2/SLC2A* and fatty acid binding proteins 2 (*FABP2/I-FABP*)) and inflammatory cytokine (*IL-1, IL-6, IL-8, IL-10, IL-12A* (*p35*), *IL-17R, TNF-α*, interferon *γ* (*IFN-γ*), transforming growth factor *β*1 (TGF-*β1*) and forkhead box P3 (*FOXP3*)) genes were designed using Primer Express^™^ (Applied Biosystems) and synthesised by MWG Biotech. Primer sequences are presented in Table 2. The nutrient transporter and inflammatory cytokine genes chosen were selected based on previous work by Heim *et al.*^(^^18^^,^^25^^)^. The efficiency of all primer sets was established using a semi-log curve of quantity *v.* control of 2-fold serial dilutions of cDNA as reported previously by Smith *et al*.^(^^31^^)^. The following porcine reference genes were used as described previously by Ryan *et al*.^(^^32^^)^: hydroxymethyl-bilane synthase (HMBS) and peptidylprolylisomerase A (*PPIA*) used for 0 h (immediately after birth) and weaning samples, and HMBS and tyrosine 3-mono-oxygenase/tryptophan 5-mono-oxygenase activation protein, zeta polypeptide (YWHAZ) used for 48 h after birth samples. The RT-qPCR was then carried out on cDNA using the ABI PRISM 7500 Fast sequence detection system for ninety-six-well plates (Applied Biosystems). All samples were prepared in duplicate using the SYBR Green Fast PCR Master Mix (Applied Biosystems), cDNA as the template and specific primers for the genes selected. For each reaction, 5 µl cDNA, 1·2 µl forward and reverse primer mix (300 nm), 3·8 µl nuclease-free water and 10 µl Fast SYBR Green PCR Master Mix were added and made up to a final volume of 20 µl. The two-step PCR programme was as follows: 95°C for 10 min for one cycle, followed by 95°C for 15 s and 60°C for 1 min for forty cycles.

The raw *C*_*t*_ values for the reference genes were converted to relative quantities using the formula *Q* = *E*^Δ^*C*_*t*_ where *E* is the PCR efficiency of the assay and ^Δ^*C*_*t*_ is the value calculated for the difference between the lowest *C*_*t*_ value and the *C*_*t*_ value of the sample in question for each gene. The relative quantities of the endogenous controls were then analysed for stability in geNorm^(^^33^^)^. The stability ‘*M*’ value generated by the geNorm application for the selected endogenous controls (β-actin (*ACTB*), glyceraldehyde-3-phosphate dehydrogenase (*GAPDH*) and *PPIA*) which was less than 1·5 indicated their suitability as endogenous controls for these intestinal samples. The geometric mean of the relative quantities for *HMBS, YWHAZ* and *PPIA* (normalisation factor) was then calculated using geNorm. The relative quantities were divided by the normalisation factor (obtained in geNorm) for that sample to give the final normalised relative expression for each target gene.

### Statistical analysis

All data were analysed by using the General Linear Model procedure of SAS^(^^34^^)^. All data were initially checked for normality using the Univariate procedure in SAS^(^^34^^)^. Inflammatory cytokine and nutrient transporter gene expression in ileum tissue, small-intestinal morphology and *Lactobacillus* spp. and Enterobacteriaceae quantification data were analysed as a complete randomised design, with the piglet (sow pen) as the experimental unit. GCN of *Lactobacillus* spp. and Enterobacteriaceae were log-transformed before statistical analysis. Inflammatory cytokine gene expression in colon tissue data was analysed as a 2 × 2 factorial arrangement, with the piglet (sow pen) as the experimental unit. The statistical model used included the effects of maternal dietary treatment and challenge (LPS or PBS), and their associated two-way interactions. The sow lactation data were analysed as a complete randomised design, with the sow pen as the experimental unit. For piglet diarrhoea score, data were analysed by repeated-measures analysis using the PROC MIXED procedure of SAS. The model included the effects of maternal dietary treatment and time (days after birth) and the associated two-way interactions. The pdiff function of SAS was used to separate means. Simple regression models were used to determine the relationships between BW and small-intestinal morphology, between small-intestinal morphology and nutrient transporter gene expression, and between inflammatory cytokine and nutrient transporter gene expression. Animal (piglet) was modelled as a random effect. All data presented in the tables are expressed as least-square means with their standard errors. The probability level that denotes significance is *P* < 0·05, a numerical trend is *P* > 0·05 and *P* < 0·10, and not significant *P* > 0·10.

**Table 2.** Porcine oligonucleotide primers used for real-time PCR

Gene	Accession no.	Primer (5′ 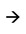 3′)	Product length (bp)
*IL-1*	NM_214029.1	F: CAGCCAACGGGAAGATTCTG	77
		R: AATGGCTTCCAGGTCGTCAT	
*IL-6*	NM_214399.1	F:GACAAAGCCACCACCCCTAA	69
		R:CTCGTTCTGTGACTGCAGCTTATC	
*IL-8*	NM_213867.1	F:TGCACTTACTCTTGCCAGAACTG	82
		R:CAAACTGGCTGTTGCCTTCTT	
*IL-10*	NM_214041.1	F:GCCTTCGGCCCAGTGAA	71
		R:AGAGACCCGGTCAGCAACAA	
*IL-12A* (*p35*)	NM_213993.1	F:CGTGCCTCGGGCAATTATA	68
		R:CGCAGGTGAGGTCGCTAGTT	
*IL-17R*	NM_001005729.1	F:CAAGCGGTGGCGTTTTGCCT	57
		R:GTCTCCGTCGGGGATGGGCT	
*IFN-γ*	NM_213948.1	F:TCTAACCTAAGAAAGCGGAAGAGAA	81
		R:TTGCAGGCAGGATGACAATTA	
*TGF-β1*	NM_214015.1	F:AGGGCTACCATGCCAATTTCT	101
		R:CGGGTTGTGCTGGTTGTACA	
*TNF-α*	NM_214022.1	F:TGGCCCCTTGAGCATCA	68
		R:CGGGCTTATCTGAGGTTTGAGA	
*FOXP3*	NM_001128438.1	F:GTGGTGCAGTCTCTGGAACAAC	68
		R:AGGTGGGCCTGCATAGCA	
*PPIA*	NM_214353.1	F: CGGGTCCTGGCATCTTGT	75
		R: TGGCAGTGCAAATGAAAAACTG	
*HMBS*	NM_001097412.1	F: CTGAACAAAGGTGCCAAGAACA	74
		R: GCCCCGCAGACCAGTTAGT	
*YWHAZ*	XM_001927228.1	F: GGACATCGGATACCCAAGGA	71
		R: AAGTTGGAAGGCCGGTTAATTT	

F, forward; R, reverse; *IFN-γ*, interferon *γ*; TGF-*β1*, transforming growth factor *β*1; *FOXP3*, forkhead box P3; *PPIA*, peptidylprolylisomerase A; HMBS, hydroxymethyl-bilane synthase; *YWHAZ*, tyrosine 3-mono-oxygenase/tryptophan 5-mono-oxygenase activation protein, zeta polypeptide.

## Results

### Sow reproductive performance, colostrum IgG concentration and suckling piglet growth performance

Gilts offered diet supplemented with SDP had a longer gestation period compared with the basal-fed gilts (114·5 *v.* 113·5 (se 0·27) d; *P* < 0·05). Colostrum IgG concentration did not differ between maternal dietary treatments; however, it did tend towards significance (57·0 *v*. 92·4 (se 16·9) mg/ml, for basal-fed and SDP-supplemented sows, respectively; *P* = 0·07). Litter size (14·4 (se 0·72) piglets) and the number of live born piglets (12·9 (se 0·62) piglets) were not influenced by maternal dietary treatments (*P* > 0·10). Piglet BW at birth (1·76 (se 0·66) kg) and weaning (8·33 (se 0·22) kg), and average daily gain from birth to weaning (0·24 (se 0·07) kg/d) (*P* > 0·10) did not differ between sow dietary treatments.

### Diarrhoea score

**Fig. 1.**
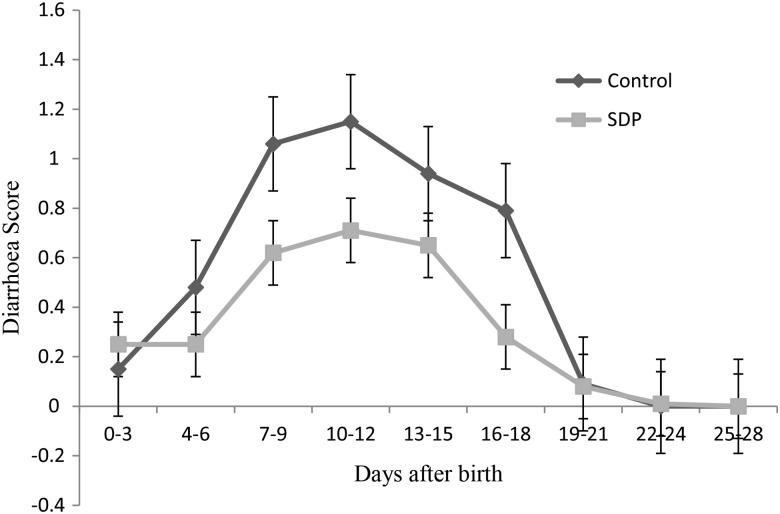
Differences in piglet diarrhoea score over time during days 0–3, 4–6, 7–9, 10–12, 13–15, 16–18, 19–21, 22–24 and 25–28 d post-birth. Diarrhoea score is measured on a scale from 0 to 3: (0) no diarrhoea; (1) slight; (2) middle; (3) acute^(^^24^^)^. (■), Group supplemented with seaweed-derived polysaccharides (SDP) containing laminarin and fucoidan; (♦), control group. Values are means, with standard errors represented by vertical bars. There were SDP (*P* = 0·010) and time (*P* < 0·001) effects. There was no SDP × time interaction (*P* > 0·10).

There was a day effect on piglet diarrhoea score (*P* < 0·05; Fig. 1). Piglets suckling the SDP-supplemented sows had a lower diarrhoea score during the lactation period compared with those suckling the basal-fed sows (*P* < 0·05; Fig. 1).

### Sow faecal and piglet colonic microbiota population

Sows supplemented with SDP had reduced Enterobacteriaceae log GCN/g of faeces on the expected parturition day compared with those fed the basal diet (*P* < 0·05; Table 3). Sow *Lactobacillus* spp. log GCN/g of faeces did not differ between dietary treatments (*P* > 0·10; Table 3).

**Table 3.** Effect of maternal dietary treatment on selected microbiota gene numbers (Least-square mean values with their standard errors)

	Control	SDP	sem	*P*
Sows – expected farrowing date (log GCN/g faeces)*				
*Lactobacillus* spp.	8·97	8·68	0·14	0·153
Enterobacteriaceae	8·55	7·76	0·24	0·031
LER	1·06	1·14	0·03	0·102
0 h – immediately after birth (log GCN/g colonic digesta)†				
*Lactobacillus* spp.	6·91	7·01	0·12	0·591
Enterobacteriaceae	7·59	7·87	0·14	0·204
LER	0·91	0·89	0·01	0·342
48 h after birth (log GCN/g colonic digesta)†				
*Lactobacillus* spp.	9·53	8·58	0·40	0·119
Enterobacteriaceae	10·01	10·34	0·30	0·453
LER	0·96	0·84	0·05	0·122
Weaning (log GCN/g colonic digesta)†				
*Lactobacillus* spp.	9·70	9·95	0·39	0·663
Enterobacteriaceae	9·14	9·08	0·43	0·921
LER	1·08	1·11	0·07	0·748

SDP, seaweed-derived polysaccharides containing laminarin and fucoidan; GCN, gene copy number; LER, ratio of *Lactobacillus* spp. to Enterobacteriaceae.

* *n* 10 sows per treatment group.

† *n* 8 piglets per treatment group.

Piglet *Lactobacillus* spp. and Enterobacteriaceae log GCN/g of colon digesta at birth (0 h), 48 h after birth and weaning did not differ between maternal dietary treatments (*P* > 0·10; Table 3).

### Piglet small-intestinal morphology

The VH and the CD in the ileum showed a numerical increase from birth to weaning (VH average: 143 *v*. 406 µm, and CD average: 26 *v*. 126 µm at birth and weaning, respectively). Piglet small-intestinal morphology at birth and 48 h after birth did not differ between maternal dietary treatments (*P* > 0·10; Table 4). There was a positive linear effect of piglet birth weight on the VH in the ileum (*R*^2^ 0·53; *P* < 0·001) and jejunum (*R*^2^ 0·41; *P* < 0·01). There was also a positive linear effect of piglet BW at 48 h after birth on the VH in the jejunum (*R*^2^ 0·41; *P* < 0·01).

**Table 4.** Effect of maternal dietary treatment on small-intestinal morphology of piglets (Least-square mean values with their standard errors)

	Control	SDP	sem	*P*
0 h – immediately after birth*				
Duodenum				
VH (μm)	174	165	16·70	0·694
CD (μm)	24	23	1·36	0·499
VH:CD	7·3	7·3	0·71	0·999
Jejunum				
VH (μm)	186	176	16·32	0·674
CD (μm)	24	26	1·31	0·283
VH:CD	7·7	7·1	0·68	0·500
Ileum				
VH (μm)	136	154	16·71	0·465
CD (μm)	25	28	1·36	0·165
VH:CD	5·6	5·5	0·71	0·946
48 h after birth*				
Duodenum				
VH (μm)	229	235	24·70	0·871
CD (μm)	30	29	2·46	0·756
VH:CD	7·6	8·2	0·78	0·600
Jejunum				
VH (μm)	179	225	24·45	0·188
CD (μm)	37	32	2·45	0·223
VH:CD	5·3	7·0	0·77	0·129
Ileum				
VH (μm)	160	198	24·58	0·277
CD (μm)	38	38	2·47	0·880
VH:CD	4·2	5·3	0·78	0·355
Weaning*				
Duodenum				
VH (μm)	465	465	23·60	0·984
CD (μm)	136	118	11·39	0·257
VH:CD	3·7	4·1	0·30	0·353
Jejunum				
VH (μm)	317	454	23·60	<0·001
CD (μm)	93	121	11·39	0·089
VH:CD	3·6	3·8	0·30	0·707
Ileum				
VH (μm)	347	466	23·60	0·001
CD (μm)	144	108	11·39	0·032
VH:CD	2·5	4·4	0·30	<0·001

SDP, seaweed-derived polysaccharides containing laminarin and fucoidan; VH, villus height; CD, crypt depth.

* *n* 8 piglets per treatment group.

At weaning, piglets suckling the SDP-supplemented sows had increased VH in the jejunum and ileum compared with those suckling the basal-fed sows (*P* < 0·05). Piglets suckling the SDP-supplemented sows had reduced CD in the ileum compared with those suckling the basal-fed sows (*P* < 0·05). Piglets suckling the SDP-supplemented sows had an increased VH:CD ratio in the ileum compared with those suckling the basal-fed sows (*P* < 0·05). There was a positive linear effect of piglet BW at weaning on the VH in the duodenum (*R*^2^ 0·35; *P* < 0·05).

### Nutrient transporter gene expression

*GLUT1, GLUT2* and *PEPT1* are highly expressed at birth (relative abundance average: 1·38, 1·85 and 1·76 for *GLUT1, GLUT2* and *PEPT1*, respectively), followed by a decline to the end of lactation (relative abundance average: 0·65, 0·41 and 0·43 for *GLUT1, GLUT2* and *PEPT1,* respectively). The gene expression of *FAPB2* and *SGLT1* was lowly expressed at birth (relative abundance average: 1·04 and 0·83 for *FABP2* and *SGLT1*, respectively), followed by an increase to the end of lactation (relative abundance average: 1·91 and 1·79 for *FABP2* and *SGLT1*, respectively).

Nutrient transporter gene expression in ileum tissue of piglets at birth did not differ between maternal dietary treatments (*P* > 0·10; Table 5). At 48 h after birth, the gene expression of *SGLT1* was down-regulated in the ileum of piglets suckling the SDP-supplemented sows compared with those suckling the basal-fed sows (*P* < 0·05; Table 5).

**Table 5.** Effect of maternal dietary treatment on the normalised relative abundance of nutrient transporter gene expression in ileal tissue of piglets (Least-square mean values with their standard errors)

	Control	SDP	sem	*P*
0 h – immediately after birth*				
*PEPT1*	1·68	1·86	0·25	0·626
*FABP2*	0·97	1·12	0·25	0·676
*SGLT1*	0·92	0·74	0·10	0·253
*GLUT1*	1·41	0·99	0·25	0·255
*GLUT2*	1·97	1·71	0·38	0·646
48 h after birth*				
*PEPT1*	1·79	2·00	0·27	0·593
*FABP2*	1·12	1·27	0·30	0·732
*SGLT1*	1·12	0·80	0·11	0·050
*GLUT1*	1·73	1·38	0·38	0·536
*GLUT2*	2·18	1·78	0·40	0·501
Weaning*				
*PEPT1*	1·71	0·43	0·35	0·025
*FABP2*	1·78	2·05	0·65	0·783
*SGLT1*	1·42	2·23	0·44	0·226
*GLUT1*	0·98	0·65	0·06	0·003
*GLUT2*	1·76	0·41	0·36	0·025

SDP, seaweed-derived polysaccharides containing laminarin and fucoidan; *PEPT*, peptide transporter; *FABP*, fatty acid binding protein; *SGLT*, sodium–glucose-linked transporter; *GLUT*, glucose transporter.

* *n* 8 piglets per treatment group.

At weaning, the gene expression of *PEPT1, GLUT1* and *GLUT2* was down-regulated in the ileum of piglets suckling the SDP-supplemented sows compared with those suckling the basal-fed sows (*P* < 0·05; Table 5). There was a negative linear effect of VH on *GLUT1* gene expression in the ileum of piglets (*R*^2^ 0·36; *P* < 0·05).

### Inflammatory cytokine gene expression in the ileum

Inflammatory cytokine gene expression in ileum tissue of piglets at birth and 48 h after birth did not differ between maternal dietary treatments (*P* > 0·10; Table 6).

**Table 6.** Effect of maternal dietary treatment on piglet ileal transcriptional response of genes related to the immune response (Least-square mean values with their standard errors)

	Control	SDP	sem	*P*
0 h – immediately after birth*				
*FOXP3*	1·23	1·81	0·23	0·108
*IFN-γ*	1·09	1·62	0·18	0·102
*IL-1*	0·92	0·90	0·13	0·907
*IL-6*	1·19	1·17	0·18	0·926
*IL-8*	4·71	4·33	0·42	0·598
*IL-10*	1·43	2·09	0·32	0·173
*IL-12A(p35)*	0·76	1·02	0·09	0·101
*IL-17R*	0·45	0·51	0·05	0·476
*TGF-β1*	1·18	1·50	0·18	0·210
*TNF-α*	2·02	2·86	0·44	0·210
48 h after birth*				
*FOXP3*	1·03	1·59	0·39	0·329
*IFN-γ*	1·45	1·39	0·47	0·934
*IL-1*	1·31	0·97	0·45	0·609
*IL-6*	1·60	1·85	0·47	0·713
*IL-8*	2·04	2·81	0·77	0·491
*IL-10*	1·58	1·64	0·36	0·902
*IL-12A*(*p35*)	1·19	1·15	0·22	0·911
*IL-17R*	1·46	1·00	0·22	0·156
*TGF-β1*	1·46	1·24	0·30	0·605
*TNF-α*	1·22	1·69	0·30	0·288
Weaning*				
*FOXP3*	1·24	1·73	0·17	0·101
*IFN-γ*	0·91	3·22	0·37	0·001
*IL-1*	0·99	2·85	0·16	<0·001
*IL-6*	1·12	0·64	0·09	0·003
*IL-8*	1·15	0·77	0·13	0·050
*IL-10*	0·82	0·29	0·08	<0·001
*IL-12A* (*p35*)	0·97	1·93	0·13	<0·001
*IL-17R*	1·16	0·35	0·11	<0·001
*TGF-β1*	1·07	2·23	0·13	<0·001
*TNF-α*	0·93	1·66	0·07	<0·001

SDP, seaweed-derived polysaccharides containing laminarin and fucoidan; *FOXP3*, forkhead box P3; *IFN-γ*, interferon *γ*; TGF-*β1*, transforming growth factor *β*1.

* *n* 8 piglets per treatment group.

There was a positive linear effect of piglet birth weight on *IL-10* gene expression in the ileum (*R*^2^ 0·41; *P* < 0·01). There was a negative linear effect of *TNF-α* gene expression on *GLUT1* gene expression in the ileum of piglets at birth (0 h) (*R*^2^ < 0·31; *P* < 0·05). There was a negative linear effect of piglet BW at 48 h after birth on *TNF-α* (*R*^2^ 0·36; *P* < 0·05) and *IL-10* (*R*^2^ < 0·38; *P* < 0·01) gene expression in the ileum.

At weaning, the gene expression of *IL-1, IL-12A* (*p35*), *IFN-γ, TGF-β1* and *TNF-α* was up-regulated and the gene expression of *IL-10, IL-17R, IL-6* and *IL-8* was down-regulated in the ileum of piglets suckling the SDP-supplemented sows compared with those suckling the basal-fed sows (*P* < 0·05; Table 6). There was a negative linear effect of *TNF-α* gene expression on *GLUT1* (*R*^2^ 0·51; *P* < 0·01), *GLUT2* (*R*^2^ 0·41; *P* < 0·01) and *PEPT1* (*R*^2^ 0·43; *P* < 0·01) gene expression in the ileum of piglets.

### Inflammatory cytokine gene expression following an *ex vivo* lipopolysaccharide challenge in the colon

Inflammatory cytokine gene expression in piglet colon tissue following an *ex vivo* LPS challenge at birth did not differ between maternal dietary treatments (*P* > 0·10) (Table 7). The LPS challenge up-regulated the gene expression of *IL-6, IL-8, IL-10* and *TNF-α* and down-regulated the gene expression of *FOXP3, IL-1, IL-12A* (*p35*), *IL-17R, IFN-γ* and *TGF-β1* in the colon tissue of piglets at birth (*P* < 0·05) (Table 7).

**Table 7.** Effect of maternal dietary treatment on piglet colonic transcriptional response genes related to the immune response following an *ex vivo* lipopolysaccharide (LPS) challenge (Least-square mean values and pooled standard errors)

	SDP		LPS			P**	
	No	Yes	No	Yes	sem*	SDP	LPS
0 h – immediately after birth†							
*FOXP3*	0·66	0·54	0·81	0·40	0·06	0·153	<0·001
*IFN-γ*	0·61	0·69	1·06	0·25	0·13	0·676	<0·001
*IL-1*	0·95	0·93	1·14	0·74	0·08	0·876	0·004
*IL-6*	1·03	1·25	0·92	1·36	0·13	0·245	0·023
*IL-8*	0·72	0·60	0·24	1·08	0·14	0·540	<0·001
*IL-10*	1·11	1·16	0·71	1·57	0·20	0·849	0·005
*IL-12A* (*p35*)	0·99	1·13	1·31	0·80	0·13	0·482	0·013
*IL-17R*	1·80	2·01	2·13	1·68	0·12	0·218	0·014
*TGF-β1*	0·79	0·71	0·84	0·65	0·05	0·228	0·006
*TNF-α*	0·81	0·40	0·53	0·77	0·06	0·353	0·054
48 h after birth†							
*FOXP3*	1·17	1·03	1·05	1·15	0·14	0·480	0·617
*IFN-γ*	1·77	1·51	1·63	1·64	0·53	0·729	0·993
*IL-1*	2·16	1·30	1·41	2·05	0·46	0·196***	0·332
*IL-6*	2·71	1·24	1·05	2·90	0·64	0·119***	0·052
*IL-8*	1·75	0·97	1·21	1·52	0·39	0·170	0·573
*IL-10*	2·06	1·43	1·43	2·06	0·51	0·390	0·390
*IL-12A* (*p35*)	1·13	1·06	1·05	1·14	0·12	0·666	0·619
*IL-17R*	1·11	1·04	1·13	1·02	0·13	0·687	0·529
*TGF-β1*	1·13	1·06	1·08	1·11	0·11	0·628	0·873
*TNF-α*	1·51	1·30	1·40	1·41	0·29	0·619	0·990
Weaning†							
*FOXP3*	1·22	2·07	1·44	1·86	0·12	<0·001	0·027
*IFN-γ*	1·64	2·96	2·03	2·57	0·40	0·028	0·348
*IL-1*	1·24	1·06	1·42	0·88	0·22	0·574	0·101
*IL-6*	0·98	0·33	0·64	0·67	0·13	0·002	0·901
*IL-8*	1·92	1·84	0·93	2·83	0·41	0·892	0·003
*IL-10*	2·01	1·71	0·80	2·92	0·39	0·588	<0·001
*IL-12A* (*p35*)	1·38	1·47	1·18	1·67	0·27	0·811	0·207
*IL-17R*	1·20	1·00	1·01	1·19	0·10	0·199	0·240
*TGF-β1*	0·95	1·25	1·12	1·08	0·10	0·048	0·827
*TNF-α*	1·55	1·72	1·08	2·19	0·36	0·738	0·040

SDP, seaweed-derived polysaccharides containing laminarin and fucoidan; *FOXP3*, forkhead box P3; *IFN-γ*, interferon *γ*; TGF-*β1*, transforming growth factor *β*1.

† *n* 8 piglets per treatment group.

* Pooled sem.

** There was no SDP × LPS interaction (*P* > 0·10).

*** There was a SDP × LPS interaction (*P* < 0·05).

At 48 h after birth, there was an interactive effect between maternal dietary treatment and LPS challenge on the gene expression of *IL-1* and *IL-6* (*P* < 0·05) (Table 7). The gene expression of *IL-1* and *IL-6* was down-regulated in the LPS-challenged colon tissue of piglets suckling the SDP-supplemented sows compared with those suckling the basal-fed sows (*P* < 0·05). However, there was no difference in the gene expression of *IL-1* and *IL-6* in the unchallenged colon tissue between treatment groups (*P* > 0·10). The LPS challenge up-regulated the gene expression of *IL-6* in the colonic tissue of piglets at weaning (*P* < 0·05) (Table 7).

At weaning, there was no maternal dietary treatment × LPS challenge interaction on cytokine gene expression (*P* > 0·10) (Table 7). The gene expression of *FOXP3, IFN-γ* and *TGF-β1* was up-regulated and *IL-6* was down-regulated in the colon tissue of piglets suckling the SDP-supplemented sows compared with those suckling the basal-fed sows (*P* < 0·05) (Table 7). The LPS challenge up-regulated the gene expression of *FOXP3, IL-8, IL-10* and *TNF-α* in the colonic tissue of piglets at weaning (*P* < 0·05) (Table 7).

## Discussion

The hypothesis of this study is that maternal SDP supplementation from day 83 of gestation would improve piglet BW at birth and modulate selected intestinal microbial populations, the inflammatory response and aspects of intestinal health of piglets at birth and during the suckling period, making them more immune competent to deal with post-weaning adversities. The positive response observed in piglets suckling the SDP-supplemented sows, such as down-regulation of pro-inflammatory cytokine gene expression, and improved intestinal morphology in the ileum, partially supports this hypothesis. Dietary supplementation of SDP increased the gestation length. Gestation length of supplemented animals was prolonged by 1 d compared with the basal-fed gilts. The sow's gestation length is an average of 114 d, with 85 % of farrowing concentrating between 114 and 116 d of gestation^(^^35^^)^. Pig litters are unique: large litters at birth and the piglets are born relatively well developed. Much of this development takes place during the last few days of gestation^(^^36^^)^. It seems likely that prolonged gestation would improve piglet maturation at birth, as the nutrient needs for fetal growth increase from day 69 of gestation^(^^19^^)^, and the transference of nutrients to the fetus increases between days 90 and 100 of gestation^(^^20^^)^. SDP contain sugars normally not present in plant dietary fibres^(^^37^^,^^38^^)^. It was expected that these sugars, particularly laminarin, would provide nutrients to the fetus and consequently increase piglet birth weight. However, in the present study the increase in gestation length did not influence piglet BW at birth. There was also no effect of maternal dietary treatment on piglet BW at weaning. In agreement, Leonard *et al*.^(^^17^^)^ reported that sow dietary supplementation of a similar SDP from day 109 of gestation did not favour the growth performance of piglets during the lactation period.

Maternal dietary SDP supplementation numerically increased colostrum IgG concentration (*P* = 0·07). This is probably due to immunomodulatory property of laminarin^(^^39^^)^. Laminarin is absorbed from the ileum, as recent work by Heim *et al*.^(^^25^^)^ showed that there was an increase in glucose transporters in the ileum following the ingestion of laminarin in weaned pigs. According to Bourne & Curtis^(^^39^^)^, all colostral IgG is derived from the serum of the sow. The IgG uptake from serum is then mediated by specific Fc-dependent receptors on mammary epithelial cells^(^^40^^,^^41^^)^. Previously, Leonard *et al*.^(^^17^^)^ reported that maternal supplementation of a similar SDP from day 109 of gestation increased colostrum IgG concentration.

### Aspects of intestinal health and immune status

The intestine is a major site of digestion, nutrient absorption, harbouring a complex microbiota and a highly evolved mucosal immune system^(^^11^^,^^42^^)^. In the small intestine, the nutrients undergo a series of degradative steps carried out by digestive enzymes^(^^43^^,^^44^^)^ that can then be efficiently absorbed via nutrient transporters expressed along the crypt–villus axis in the enterocytes^(^^43^^)^. Under conventional practice, abrupt weaning is often associated with undesirable morphological changes in the small-intestinal architecture, which include villous atrophy and crypt hyperplasia^(^^45^^,^^46^^)^. There was no effect of maternal dietary treatment on nutrient transporter gene expression in the ileum of newborn piglets (0 h). There was also no effect of maternal dietary treatment on small-intestinal morphology. There was a positive linear effect of piglet birth weight on VH in the ileum and jejunum. Maternal dietary supplementation of SDP resulted in a down-regulation of the gene expression of *SGLT1* in the ileum of piglets at 48 h after birth, and *PEPT1, GLUT1* and *GLUT2* at weaning. It is still not clear why maternal dietary supplementation of SDP down-regulated protein and glucose transporter gene expression in the ileum of suckling piglets. First, it may be related to an increase in VH in the ileum of these piglets. According to the regression analysis, there was a negative relationship between VH and the gene expression of *GLUT1* at weaning. However, in this study, only the mRNA abundance of these nutrient transporters was measured, and unfortunately the nutrient transporter proteins were not measured. The expression of *SGLT1* is tightly linked to the villus architecture^(^^47^^)^. Regulation of glucose transport by diet may involve increased transcription of SGLT1 mainly in crypt cells. As cells migrate to the villus, the mRNA is degraded, and transporter proteins are then inserted into the membrane, leading to increases in glucose transport about 1 d after an increase in carbohydrate levels. Endocrine factors regulate the development of intestinal brush-border enzymes during weaning, but do not regulate the development of intestinal glucose transporters^(^^47^^)^. The absence of regulation of glucose transport in suckling animals that nurse on milk is due to animals consuming a diet that does not vary in carbohydrate composition^(^^48^^)^. It is possible that SGLT1 in cells present in the small intestine since birth cannot be regulated, and dietary regulation begins only when these cells are eventually replaced. The difference in SGLT1 expression may have been due to difference in milk composition. Unfortunately, milk composition was not measured in this experiment. However, Leonard *et al*.^(^^14^^)^ demonstrated that sow dietary supplementation of a similar SDP altered milk composition during lactation.

Second, the down-regulation of *PEPT1* gene expression in the ileum of piglets suckling the SDP-supplemented sows at weaning may be attributed to the up-regulation of pro-inflammatory cytokine gene expression^(^^49^^)^. This is supported by a negative relationship between *TNF-α* gene expression and *PEPT1* gene expression in the ileum at weaning. According to Shu *et al*.^(^^49^^)^, bacterial infections alter *PEPT1* expression level in the intestine. Furthermore, in the current study, a panel of pro-inflammatory cytokines was up-regulated in the ileum of piglets suckling SDP-supplemented sows at weaning. Third, the down-regulation of nutrient transporter gene expression may be attributed to the polysaccharide fucoidan. Heim *et al*.^(^^50^^)^ reported that when gilts were supplemented with fucoidan, from day 107 of gestation to weaning (day 24), the gene expression of SGLT1 was down-regulated in the ileum of piglets at weaning.

The present data also show that *GLUT1, GLUT2* and *PEPT1* are highly expressed at birth followed by a decline to the end of lactation. On the other hand, the gene expression of *FAPB2* and *SGLT1* was lowly expressed at birth, followed by an increase to the end of lactation. VH and CD in the ileum showed a numerical increase from birth to weaning. Interestingly, there was a positive linear effect of piglet BW at birth on VH in the ileum. However, no maternal dietary treatment effect was observed on piglet BW at birth and VH in the ileum. In addition, maternal dietary SDP supplementation positively changed piglet small-intestinal architecture at weaning (increased VH and VH:CD ratio). The VH:CD ratio is a useful criterion for assessing intestinal health and function^(^^46^^)^. This increase in VH and the VH:CD ratio may be attributed to the reduced diarrhoea score during the lactation period. Reduction in VH and the VH:CD ratio have been associated with increased incidences of scouring in weaned pigs challenged with enterotoxigenic *E. coli* ^(^^18^^)^.

Immediately after birth the neonatal intestine is rapidly invaded by bacteria, originating from the mother and the environment^(^^8^^)^ and this primary colonisation is important for the right development and programming of the animal's local and systemic immune system^(^^5^^)^. Dietary provision of SDP reduced dam Enterobacteriaceae GCN/g faeces on the expected parturition day, indicating an antimicrobial property of SDP. Previous research indicated that supplementation of a similar SDP to sows decreased their faecal Enterobacteriaceae numbers at parturition^(^^17^^)^. The reduction in sow Enterobacteriaceae GCN/g faeces may be attributed to the potential agglutination properties of the SDP. *β*-Glucans (laminarin) have the capacity to agglutinate certain bacterial species, thus inhibiting subsequent attachment and colonisation of epithelial mucosal surfaces^(^^51^^)^. Furthermore, fucoidan possesses numerous biological functions, including antimicrobial properties^(^^52^^)^. Demecková *et al*.^(^^4^^)^ reported that sow faecal microbiota composition at farrowing influences subsequent bacterial colonisation of the neonatal intestinal tract. In the current study, lactobacilli were selected as bacterial indicators of beneficial bacteria, while Enterobacteriaceae were chosen based on their potential association with gastrointestinal disequilibrium^(^^53^^)^. Piglets suckling the SDP-supplemented sows had reduced diarrhoea score during the lactation period. However, the decline in sow Enterobacteriaceae GCN/g faeces did not affect piglet Enterobacteriaceae GCN/g colonic digesta at birth, 48 h after birth and weaning. This reduction in diarrhoea score may be attributed to the immunomodulatory effects of SDP in enhancing the cellular and humoral immune function, as well as suppressing the *E. coli* population^(^^17^^)^. Leonard *et al*.^(^^17^^)^ demonstrated that sow SDP supplementation reduced piglet colonic *E. coli* numbers at weaning. A suppressed colonic *E. coli* number may ultimately alleviate the incidence and severity of diarrhoea. Unfortunately, *E. coli* GCN was not measured in the current study.

LPS is a component of the outer membrane of Gram-negative bacteria and a commonly used immunostimulant^(^^54^^)^. To mimic an immunological stress and the response of pigs exposed to a microbial challenge, colon tissue of piglets was LPS challenged *ex vivo* at birth, 48 h after birth and weaning. The biological effects mediated by LPS are attributed to inflammatory cytokine synthesis and release by stimulated macrophages^(^^55^^,^^56^^)^. The LPS challenge up-regulated the gene expression of a panel of pro-inflammatory cytokines at birth (*IL-6, IL-8* and *TNF-α*), 48 h after birth (*IL-6*) and weaning (*IL-8* and *TNF-α*), proving the LPS challenge worked.

The colonic tissue of piglets suckling the SDP-supplemented sows, following LPS challenge had *IL-1* and *IL-6* gene expression down-regulated at 48 h after birth. There are a number of ways these findings could be interpreted. Porcine epithelial cells respond to various pathogens with the production of TNF-α and IFN-γ which block pathogen replication and cytokines are also involved in recruitment and activation of immune cells^(^^57^^)^. The fact that piglets suckling the basal fed sows displayed increased expression of *IL-1* and *IL-6* in response to LPS suggests that these animals retained a capacity to respond more adequately to infection relative to the piglets suckling SDP-supplemented sows. An alternative hypothesis is that piglets suckling the SDP-supplemented sows possess a number of inherent energy-saving mechanisms, one of which is exhibited as a reduced inflammatory response following infection, as a result of maternal feeding of SDP. While both pig populations had no differences in their pathogenic bacteria levels at 48 h, experimental infection would be necessary to establish if this reduction in cytokine expression results in a measurable decrease in their resistance to pathogenic bacteria.

At weaning, the gene expression of the anti-inflammatory cytokine *TGF-β1* was up-regulated and the gene expression of the pro-inflammatory cytokines *IL-6* and *IL-8* was down-regulated in the ileum and colon tissues of piglets suckling the SDP-supplemented sows. *IL-6* is produced by a number of cell types including macrophages, endothelial cells, B cells and mast cells, and is up-regulated in most, if not all inflammatory states^(^^58^^)^. *IL-8* is known to be regulated by inflammatory signals^(^^59^^)^. *IL-6* and *IL-8* are cytokines classically involved in *E. coli*-caused diarrhoea^(^^60^^)^. Down-regulation of the gene expression of these cytokines may be related to the reduced faecal scores observed in piglets suckling the SDP-supplemented sows. If the maternal SDP supplementation suppresses the secretion of pro-inflammatory cytokines, this could lower the activity of the immune system resulting in an energy-saving mechanism and increased growth performance during the suckling and post-weaning periods, as suggested by Li *et al*.^(^^61^^)^.

There was a positive linear relationship between piglet BW at birth and the anti-inflammatory *IL-10* gene expression in the ileum. IL-10 is considered a potent anti-inflammatory cytokine that strongly inhibits the production of pro-inflammatory cytokines^(^^62^^)^. There was negative linear relationship between piglet BW at 48 h after birth and gene expression of the pro-inflammatory *TNF-α* in the ileum, enforcing the concept of maximising piglets’ ingestion of colostrum and milk in the first 48 h after birth.

A panel of pro-inflammatory cytokines was also up-regulated in the ileum and colon tissues of piglets suckling the SDP-supplemented sows at weaning. However, this pro-inflammatory response did not affect piglet growth performance during the lactation period. These cytokines play an important role in acute inflammation and are responsible for neutrophil recruitment and activation to the initial site of infection^(^^63^^)^. *β*-Glucans can stimulate macrophages, neutrophils and natural killer cells, and could promote T cell-specific responses by induction of pro-inflammatory cytokines like *IFN-γ, IL-8* and *IL-12A* (*p35*) from those cells^(^^64^^–^^66^^)^. This pro-inflammatory response could be of particular importance because piglets often encounter presenting pathogens immediately post-weaning. Leonard *et al*.^(^^17^^)^ reported that maternal SDP supplementation enhanced the expression of *TNF-α* in the ileum after an *ex vivo* LPS challenge at weaning.

### Conclusion

Maternal dietary SDP supplementation down-regulated the gene expression of a panel of pro-inflammatory cytokines, classically involved in *E. coli*-caused diarrhoea in piglets at 48 h after birth and weaning, indicating an important immunomodulatory effect of SDP. Maternal SDP supplementation also had positive effect in the small-intestinal architecture of suckling piglets, represented by the increased VH in the ileum of piglets at weaning. Even though little positive effect was observed in nutrient transporter gene expression and the microbiota population of suckling piglets, the results provide new insights into the protective activity of maternal SDP supplementation, making the suckling piglets more immune competent to deal with pathogens commonly encountered during the immediate post-weaning period.

## References

[1] Heim G, Mellagi APG, Bierhals T, (2011) Absorption of IgG via colostrum in biological piglets and adopted piglets after cross-fostering. Arq Bras Med Vet Zoot 63, 1073–1078.

[2] Le Dividich J, Rooke JA & Herpin P (2005) Nutritional and immunological importance of colostrum for the new-born pig. J Agric Sci 143, 469–485.

[3] Zhang H, Malo C & Buddington RK (1997) Suckling induces rapid intestinal growth and changes in brush border digestive functions of newborn pigs. J Nutr 127, 418–426. 9082025 10.1093/jn/127.3.418

[4] Demecková V, Kelly D, Coutts AG, (2002) The effect of fermented liquid feeding on the faecal microbiology and colostrum quality of farrowing sows. Int J Food Microbiol 79, 85–97. 12382688 10.1016/s0168-1605(02)00182-4

[5] Yao K, Sun Z, Liu Z, (2013) Development of the gastrointestinal tract in pigs In Nutritional and Physiological Functions of Amino Acids in Pigs, pp. 3–18 [F Blachier, G Wu and Y Yin, editors].Vienna: Springer Verlag.

[6] Maxwell CV & Carter SD (2001) Feeding the weaned pig In Swine Nutrition, 2nd ed., pp. 692–715 [AJ Lewis and LL Southern, editors]. Boca Raton, FL: CRC Press.

[7] Salmon H (1999) The mammary gland and neonate mucosal immunity. Vet Immun Immunopathol 72, 143–155. 10.1016/s0165-2427(99)00127-0 10614504

[8] Kostantinov SR, Favier CF, Zhu WY, (2004) Microbial diversity studies of the porcine gastrointestinal ecosystem during weaning transition. Anim Res 53, 317–324.

[9] Xu RJ, Wang F & Zhang SH (2000) Postnatal adaptation of the gastrointestinal tract in neonatal pigs: a possible role of milk-borne growth factors. Livest Sci 66, 95–107.

[10] Rome S, Barbot L, Windsor E, (2002) The regionalization of PepT1, NBAT and EAAC1 transporters in the small intestine of rats are unchanged from birth to adulthood. J Nutr 132, 1009–1011. 11983829 10.1093/jn/132.5.1009

[11] Takanashi N, Tomosada Y, Villena J, (2013) Advanced application of bovine intestinal epithelial cell line for evaluating regulatory effect of lactobacilli against heat-killed enterotoxigenic *Escherichia coli*-mediated inflammation. BMC Microbiol 13, 54. 23497067 10.1186/1471-2180-13-54 PMC3605377

[12] Cario E, Brown D, McKee M, (2002) Commensal-associated molecular patterns induce selective Toll-like receptor trafficking from apical membrane to cytoplasmic compartments in polarized intestinal epithelium. Am J Pathol 160, 165–173. 11786410 10.1016/S0002-9440(10)64360-X PMC1867149

[13] Dibner JJ & Richards JD (2005) Antibiotic growth promoters in agriculture: history and mode of action. Poult Sci 84, 634–643. 15844822 10.1093/ps/84.4.634

[14] Leonard SG, Sweeney T, Bahar B, (2010) Effect of maternal fish oil and seaweed extract supplementation on colostrum and milk composition, humoral immune response, and performance of suckled piglets. J Anim Sci 88, 2988–2997. 20453086 10.2527/jas.2009-2764

[15] Leonard SG, Sweeney T, Pierce KM, (2010) The effects of supplementing the diet of the sow with seaweed extracts and fish oil on aspects of gastrointestinal health and performance of the weaned piglet. Br J Nutr 105, 549–560.20875191

[16] Leonard SG, Sweeney T, Bahar B, (2011) Effects of dietary seaweed extract supplementation in sows and post-weaned pigs on performance, intestinal morphology, intestinal microflora and immune status. Br J Nutr 106, 688–699. 21736851 10.1017/S0007114511000997

[17] Leonard SG, Sweeney T, Bahar B, (2012) Effect of maternal seaweed extract supplementation on suckling piglet growth, humoral immunity, selected microflora, and immune response after an *ex vivo* lipopolysaccharide challenge. J Anim Sci 90, 505–514. 21948611 10.2527/jas.2010-3243

[18] Heim G, Sweeney T, O'Shea CJ, (2014) Effect of maternal supplementation with seaweed extracts on growth performance and aspects of gastrointestinal health of newly weaned piglets after challenge with enterotoxigenic *Escherichia coli* K88. Br J Nutr 112, 1955–1965. 25345748 10.1017/S0007114514003171

[19] McPherson RL, Ji F, Wu G, (2004) Growth and compositional changes of fetal tissues in pigs. J Anim Sci 82, 2534–2540. 15446468 10.2527/2004.8292534x

[20] Biensen NJ, Wilson ME & Ford SP (1998) The impact of either a Meishan or Yorkshire uterus on Meishan or Yorkshire fetal and placental development to days 70, 90, and 110 of gestation. J Anim Sci 76, 2169–2176. 9734868 10.2527/1998.7682169x

[21] Lynch MB, Sweeney T, Callan JJ, (2009) The effect of dietary *Laminaria*-derived laminarin and fucoidan on nutrient digestibility, nitrogen utilisation, intestinal microflora and volatile fatty acid concentration in pigs. J Sci Food Agric 90, 430–437. 20355064 10.1002/jsfa.3834

[22] National Research Council (2012) Nutrient Requirements of Swine, 11th ed. Washington, DC: National Academies Press.

[23] Heim G, Mellagi APG, Bierhals T, (2012) Effects of cross-fostering within 24 h after birth on pre-weaning behaviour, growth performance and survival rate of biological and adopted piglets. Livest Sci 150, 121–127.

[24] Stamati S, Alexopoulos C, Siochu A, (2006) Probiosis in sows by administration of *Bacillus toyoi* spores during late pregnancy and lactation: effect on their health status/performance and on litter characteristics. Intern J Probiotics Prebiotics 1, 33–40.

[25] Heim G, Walsh AM, Sweeney T, (2014) Effect of seaweed derived laminarin and fucoidan, and zinc oxide on gut morphology, nutrient transporters, nutrient digestibility, growth performance and selected microbial populations in weaned pigs. Br J Nutr 111, 1577–1585. 24502994 10.1017/S0007114513004224

[26] Usov AI, Smirnova GP & Klochkova NG (2001) Polysaccharides of algae: 55. Polysaccharide composition of several brown algae from Kamchatka. Russ J Bioorg Chem 27, 395–399. 10.1023/a:1012992820204 11811067

[27] O'Shea CJ, Sweeney T, Bahar B, (2012) Indices of gastrointestinal fermentation and manure emissions of growing–finishing pigs as influenced through singular or combined consumption of *Lactobacillus plantarum* and inulin. J Anim Sci 90, 3848–3857. 22859763 10.2527/jas.2011-4461

[28] Metzler-Zebeli BU, Hooda S, Pieper R, (2010) Nonstarch polysaccharides modulate bacterial microbiota, pathways for butyrate production, and abundance of pathogenic *Escherichia coli* in the pig gastrointestinal tract. Appl Environ Microbiol 76, 3692–3701. 20382813 10.1128/AEM.00257-10 PMC2876444

[29] Lee C, Kim J, Shin SG, (2006) Absolute and relative qPCR quantification of plasmid copy number in *Escherichia coli*. J Biotechnol 123, 273–280. 16388869 10.1016/j.jbiotec.2005.11.014

[30] Liu P, Piao XS, Thacker PA, (2010) Chito-oligosaccharide reduces diarrhea incidence and attenuates the immune response of weaned pigs challenged with *Escherichia coli* K88. J Anim Sci 88, 3871–3879. 20656977 10.2527/jas.2009-2771

[31] Smith AG, O'Doherty JV, Reilly P, (2011) The effects of laminarin derived from *Laminaria digitata* on measurements of gut health: selected bacterial populations, intestinal fermentation, mucin gene expression and cytokine gene expression in the pig. Br J Nutr 105, 669–677. 21251335 10.1017/S0007114510004277

[32] Ryan MT, Collins CB, O'Doherty JV, (2010) Selection of stable reference genes for quantitative real-time PCR in porcine gastrointestinal tissues. Livest Sci 133, 42–44.

[33] Vandesompele J, De Preter K, Pattyn F, (2002) Accurate normalization of real-time quantitative RT-PCR data by geometric averaging of multiple internal control genes. Genome Biol 3, research0034.1–research0034.11. 10.1186/gb-2002-3-7-research0034 PMC126239 12184808

[34] Statistical Analysis Systems Institute (1985) Statistical Analysis Systems, 6.12 ed. Cary, NC: SAS Institute, Inc.

[35] Meredith MJ (1995) Pigs breeding and infertility In Animal Breeding and Infertility, pp. 278–353 [MJ Meredith, editor]. Oxford: Blackwell Science.

[36] Hakkarainen J (1975) Developmental changes of protein, RNA, DNA, lipid, and glycogen in the liver, skeletal muscle and brain of the pig. Acta Vet Scand 59, Suppl., 1–198. 1064328

[37] Lahaye M & Keaffer B (1997) Seaweed dietary fibres: structure, physico-chemical and biological properties relevant to intestinal physiology. Sci Aliments 17, 563–584.

[38] MacArtain P, Gill CIR, Brooks M, (2007) Nutritional value of edible seaweeds. Nutr Rev 65, 535–543. 18236692 10.1301/nr.2007.dec.535-543

[39] Bourne FJ & Curtis J (1973) The transfer of immunoglobins IgG, IgA and IgM from serum to colostrum and milk in the sow. Immunology 24, 157–162. 4685370 PMC1422885

[40] Huang SC, Hu Z, Hasler-Rapacz J, (1992) Preferential mammary storage and secretion of immunoglobulin γ (IgG) subclasses in swine. J Reprod Immunol 21, 15–28. 1734075 10.1016/0165-0378(92)90037-5

[41] Schnulle PM & Hurley WL (2003) Sequence and expression of the FcRn in the porcine mammary gland. Vet Immunol Immunopathol 91, 227–231. 12586485 10.1016/s0165-2427(02)00294-5

[42] Lallès JP, Bosi P, Smidt H, (2007) Weaning – a challenge to gut physiologists. Livest Sci 108, 82–93.

[43] Daniel H (2004) Molecular and integrative physiology of intestinal peptide transport. Annu Rev Physiol 66, 361–384. 14977407 10.1146/annurev.physiol.66.032102.144149

[44] Van Goudoever JB, Corpeleijn W, Riedijk M, (2008) The impact of enteral insulin-like growth factor 1 and nutrition on gut permeability and amino acid utilization. J Nutr 138, 1829S–1833S. 18716194 10.1093/jn/138.9.1829S

[45] Cera KR, Mahan DC, Cross RF, (1988) Effect of age, weaning and postweaning diet on small intestinal growth and jejunal morphology in young swine. J Anim Sci 66, 574–584. 3372395 10.2527/jas1988.662574x

[46] Pluske JR, Hampson DJ & Williams IH (1997) Factors influencing the structure and function of the small intestine in the weaned pig: a review. Livest Prod Sci 51, 215–236.

[47] Ferraris RP (2001) Dietary and developmental regulation of intestinal sugar transport. Biochem J 360, 265–276. 11716754 10.1042/0264-6021:3600265 PMC1222226

[48] Toloza EM & Diamond J (1992) Ontogenetic development of nutrient transporters in rat intestine. Am J Physiol 263, G593–G604. 1443134 10.1152/ajpgi.1992.263.5.G593

[49] Shu HJ, Takeda H, Shinzawa H, (2002) Effect of lipopolysaccharide on peptide transporter 1 expression in rat small intestine and its attenuation by dexamethasone. Digestion 65, 21–29. 11961339 10.1159/000051927

[50] Heim G, O'Shea CJ, Doyle DN, (2015) Effect of maternal dietary supplementation of laminarin and fucoidan, independently or in combination, on pig growth performance and aspects of intestinal health. Anim Feed Sci Tech 204, 28–41.

[51] Sweeney T, Collins C, Reilly P, (2012). Effect of purified β-glucans derived from *Laminaria digitata, Laminaria hyperborea* and *Saccharomyces cerevisiae* on piglet performance, selected bacterial populations, volatile fatty acids and pro-inflammatory cytokines in the gastrointestinal tract of pigs. Br J Nutr 108, 1226–1234. 22313684 10.1017/S0007114511006751

[52] Shibata H, Iimuro M, Uchiya N, (2003) Preventive effects of *Cladosiphon* fucoidan against *Helicobacter pylori* infection in Mongolian gerbils. Helicobacter 8, 59–65. 12603617 10.1046/j.1523-5378.2003.00124.x

[53] Nyachoti CM, Omogbenigun FO, Rademacher M, (2006). Performance responses and indicators of gastrointestinal health in early-weaned pigs fed low-protein amino acid-supplemented diets. J Anim Sci 84, 125–134. 16361499 10.2527/2006.841125x

[54] Medvedev AE, Piao W, Shoenfelt J, (2007) Role of TLR4 tyrosine phosphorylation in signal transduction and endotoxin tolerance. J Biol Chem 282, 16042–16053. 17392283 10.1074/jbc.M606781200 PMC2675888

[55] Johnson RW & von Borell E (1994) Lipopolysaccharide-induced sickness behavior in pigs is inhibited by pretreatment with indomethacin. J Anim Sci 72, 309–314. 8157515 10.2527/1994.722309x

[56] Johnson RW (1997) Inhibition of growth by pro-inflammatory cytokines: an integrated view. J Anim Sci 75, 1244–1255. 9159271 10.2527/1997.7551244x

[57] Mair KH, Sedlak C, Käser T, (2014) The porcine innate immune system: an update. Dev Comp Biol 45, 321–343. 10.1016/j.dci.2014.03.022 PMC7103209 24709051

[58] Dalrymple SA, Slattery R, Aud DM, (1996) Interleukin-6 is required for a protective immune response to systemic *Escherichia coli* infection. Infec Immun 64, 3231–3235. 8757858 10.1128/iai.64.8.3231-3235.1996 PMC174212

[59] Kasahara T, Mukaida N, Yamashita K, (1991) IL-1 and TNF-α induction of IL-8 and monocyte chemotactic and activating factor (MCAF) mRNA expression in a human astrocytoma cell line. Immunology 74, 60–67. 1937574 PMC1384672

[60] McLamb BL, Gibson AJ, Overman EL, (2013) Early weaning stress in pigs impairs innate mucosal immune responses to enterotoxigenic *E. coli* challenge and exacerbates intestinal injury and clinical disease. PLOS ONE 8, e27. 10.1371/journal.pone.0059838 PMC3634819 23637741

[61] Li J, Li DF, Xing JJ, (2006) Effects of β-glucans extracted from *Saccharomyces cerevisiae* on growth performance, and immunological and somatotropic responses of pigs challenged with *Escherichia coli* lipopolysaccharide. J Anim Sci 84, 2374–2381. 16908640 10.2527/jas.2004-541

[62] Lauw FN, Pajkrt D, Hack CE, (2000) Proinflammatory effects of IL-10 during human endotoxemia. J Immunol 165, 2783–2789. 10946310 10.4049/jimmunol.165.5.2783

[63] Girard F, Oswald IP, Taranu I, (2005) Host immune status influences the development of attaching and effacing lesions in weaned pigs. Infect Immun 73, 5514–5523. 16113267 10.1128/IAI.73.9.5514-5523.2005 PMC1231136

[64] Brown GD & Gordon S (2005) Immune recognition of fungal β-glucans. Cell Microbiol 7, 471–479. 15760447 10.1111/j.1462-5822.2005.00505.x

[65] Xiao Z, Trincado CA & Murtaugh MPB (2004) Glucan enhancement of T cell IFN-γ response in swine. Vet Immunol Immunopathol 102, 315–320. 15507314 10.1016/j.vetimm.2004.09.013

[66] Chaung HC, Huang TC, Yu JH, (2009) Immunomodulatory effects of β-glucans on porcine aveolar macrophages and bone marrow haematopoietic cell-derived dendritic cells. Vet Immunol Immunopathol 131, 147–157. 19410299 10.1016/j.vetimm.2009.04.004

[67] Sauvant D, Perez J-M & Tran G (editors) (2004) Tables of Composition and Nutritional Value of Feed Materials. Pigs, Poultry, Cattle, Sheep, Goats, Rabbits, Horses, Fish. Wageningen: Wageningen Academic Publishers.

